# Intrinsic genetic characteristics determine tumor-modifying capacity of fibroblasts: matrix metalloproteinase-3 5A/5A genotype enhances breast cancer cell invasion

**DOI:** 10.1186/bcr1775

**Published:** 2007-10-08

**Authors:** Deborah L Holliday, Simon Hughes, Jacqueline A Shaw, Rosemary A Walker, J Louise Jones

**Affiliations:** 1 Centre for Tumour Biology, Institute of Cancer and CR-UK Clinical Centre, Bart's and The London, Queen Mary's School of Medicine and Dentistry, John Vane Science Centre, Charterhouse Square, London EC1M 6BQ, UK; 2 Breast Cancer Research Unit, Department of Cancer Studies and Molecular Medicine, University of Leicester, Robert Kilpatrick Clinical Sciences Building, Leicester LE1 7LX, UK

## Abstract

**Background:**

Stromal fibroblasts can contribute to tumor invasion through the release of matrix metalloproteinases (MMPs). Population studies have suggested that single nucleotide polymorphisms (SNPs) in MMP genes influence levels of expression and may be associated with breast cancer risk and with disease progression. This study directly examined the impact of MMP SNP genotype on the ability of host fibroblasts to promote tumor cell invasion.

**Methods:**

Primary breast fibroblasts were isolated from patients with (*n *= 13) or without (*n *= 19) breast cancer, and their ability to promote breast cancer cell invasion was measured in *in vitro *invasion assays. Fibroblast invasion-promoting capacity (IPC) was analyzed in relation to donor type (tumor or non-tumor patient), *MMP-1*, *MMP-3*, and *MMP-9 *SNP genotype and MMP activity using independent samples *t *test and analysis of variance. All statistical tests were two-sided.

**Results:**

Tumor-derived fibroblasts promoted higher levels of invasion than normal fibroblasts (*p *= 0.041). When IPC was related to genotype, higher levels of IPC were generated by tumor fibroblasts with the high-expressing *MMP-3 *5A/5A genotype compared with the 5A/6A and 6A/6A genotypes (*p *= 0.05 and 0.07, respectively), and this was associated with enhanced MMP-3 release. The functional importance of MMP-3 was demonstrated by enhanced invasion in the presence of recombinant MMP-3, whereas reduction occurred in the presence of a specific MMP-3 inhibitor. An inverse relationship was demonstrated between fibroblast IPC and the high-expressing *MMP-1 *genotype (*p *= 0.031), but no relationship was seen with *MMP-9 *SNP status. In contrast, normal fibroblasts showed no variation in IPC in relation to MMP genotype, with *MMP-3 *5A/5A fibroblasts exhibiting significantly lower levels of IPC than their tumor-derived counterparts (*p *= 0.04).

**Conclusion:**

This study has shown that tumor-derived fibroblasts exhibit higher levels of IPC than normal fibroblasts and that the *MMP-3 *5A/5A genotype contributes to this through enhanced MMP-3 release. Despite a high-expressing genotype, normal fibroblasts do not exhibit higher IPC or enhanced MMP release. This suggests that more complex changes occur in tumor-derived fibroblasts, enabling full expression of the MMP SNP genotype and these possibly are epigenetic in nature. The results do suggest that, in women with breast cancer, a high-expressing *MMP-3 *genotype may promote tumor progression more effectively.

## Introduction

The microenvironment plays a central role in controlling both normal and transformed cell function as well as normal tissue integrity [1]. Both *in vitro *studies and animal models have demonstrated the importance of interactions with the microenvironment in modulating tumor progression [2]. Restoration of normal microenvironment signalling has been shown to revert features of the malignant phenotype, even when the tumor cells retain their mutations [3,4]. Inflammatory cells, endothelial cells, and stromal fibroblasts all are implicated in this activity. However, a number of studies have indicated a dominant role for fibroblasts in modulating epithelial cell function, in promoting tumor cell progression, and even in initiating epithelial cell tumorigenesis [5-10].

Differences in the pattern of gene and protein expression have been identified between peri-tumoral fibroblasts and their normal counterparts [2,11]. Such differences generally are regarded as a stromal response to tumor-derived signals, which then influence the tumor-promoting activity of the stroma. For example, tumor-derived transforming growth factor-beta induces stromal hepatocyte growth factor which then, in a paracrine fashion, promotes tumor cell invasion [12].

Emerging evidence also suggests that stroma may undergo independent genetic and epigenetic modifications [13-16]. Although the functional implications of these alterations have not been established fully, such changes may be expected to contribute to altered stromal cell function and the generation of a permissive tumor microenvironment. In contrast to such acquired genetic alterations, the potential functional impact of intrinsic genetic differences in stromal populations has received less attention. A number of reports have indicated that non-tumor fibroblasts from women with breast cancer differ from those isolated from women without breast cancer [17,18]. It has been suggested that this could be an important factor in defining the diversity of tumor behavior between individuals [14].

One way in which breast fibroblasts influence tumor behavior is through the release and activation of matrix metalloproteinases (MMPs) [19]. Several MMPs have been implicated as having a role in breast cancer, including MMP-1, MMP-2, MMP-3, and MMP-9, among others [20-22]. Common functional promoter single nucleotide polymorphisms (SNPs) have been described in some of these genes in which they have the potential to influence levels of gene expression [23,24]. In *MMP-1*, the polymorphism is characterized by an insertion (2G) or deletion (1G) of a single guanine residue at -1607 base pairs (bp). The insertion of a G residue in the 2G allele forms a binding site for the Ets-1 transcription factor which results in increased transcription and MMP-1 production [25]. A single adenine insertion (6A) or deletion (5A) located at position -1171 bp in the *MMP-3 *promoter region influences MMP-3 expression by changing the affinity for repressor binding, with the 6A allele sequence having a stronger recognition for the repressor binding site [23]. This also leads to enhanced transcriptional levels and local MMP-3 production in the presence of the 5A allele [19]. In the *MMP-9 *gene, a C-to-T transition at -1306 bp results in an approximately 1.5-fold higher promoter activity compared with the C allele [26].

Many studies have examined the association between epithelial malignancy and MMP SNP status. Some have reported an increased risk for breast cancer in women carrying the *MMP-3 *5A allele [27,28], but this has not been confirmed in other studies [29,30]. In others, the *MMP-1 *2G/2G genotype has been associated with increased susceptibility to colorectal cancer [20], though not to breast cancer. To date, as far as we are aware, *MMP-9 *SNP status has not been related to cancer susceptibility.

Although the evidence for a role of MMP SNPs in enhancing susceptibility to breast cancer is somewhat equivocal, MMPs have an established role in promoting tumor cell invasion [19,31]. Thus, mechanisms leading to higher expression of MMP might be expected to enhance tumor progression. To date, no studies have investigated the influence of MMP SNP genotype on the ability of the stromal environment to modulate tumor behavior. This study has analyzed the ability of primary breast fibroblasts, from patients with or without breast cancer, to promote breast tumor cell invasion and has investigated the relationship between MMP SNP status, donor group, and fibroblast invasion-promoting capacity (IPC) by examining the contribution of MMP activity to the invasive process.

## Materials and methods

### Breast tissue and cell lines

Breast tissue was obtained from women undergoing surgery for breast carcinoma (*n *= 13; age range 37 to 85 years) or reduction mammoplasty (*n *= 19; age range 19 to 54 years) following informed consent and approval of the study by the North East London Ethics Committee and the Leicestershire and Rutland Ethics Committee. None of the patients had received pre-operative chemotherapy. The breast cancer cell line, MDA-MB 468, and the human fetal foreskin fibroblast cell line, hfff_2_, were obtained from the American Type Culture Collection (Manassas, VA, USA).

### Isolation of primary fibroblasts

Tissue, excess to histopathological diagnosis, was selected from the breast samples and fibroblasts were isolated as described previously [32]. Briefly, the tissue was digested for 12 hours in Dulbecco's modified Eagle's medium (DMEM), 10% fetal bovine serum (FBS), 2 mM L-Glutamine, 100 IU penicillin and streptomycin, 400 IU collagenase IA, and 65 IU hyaluronidase (all reagents obtained from Sigma-Aldrich Company Ltd., Poole, Dorset, UK). Following a sedimentation step at 1 g for 30 minutes, the supernatant was removed and centrifuged, washed twice in serum-free DMEM, and then filtered through a 20-μm cell strainer (BDFalcon, San Diego, CA, USA). The filtrate was spun down and the cell pellet resuspended in DMEM with 10% FBS, penicillin and streptomycin (100 U/mL), and fungizone (2.5 μg/mL) (Gibco-BRL, now part of Invitrogen Corporation, Carlsbad, CA, USA). The cells were cultured for 24 hours, then the medium was aspirated off, and the cells were washed with DMEM (to remove any non-viable cells or contaminating red blood cells) before re-feeding with complete DMEM. All experiments, including generation of conditioned media (CM), were performed using fibroblasts that had been passaged at 70% confluency a maximum of four times after initial isolation.

### Characterization of fibroblasts

Cells plated onto poly-L-lysine-coated coverslips were stained with antibodies to vimentin (clone 384; Dako UK Ltd., Ely, Cambridgeshire, UK), cytokeratin 14 (clone LL01, gift from EB Lane, University of Dundee, UK), cytokeratin 18 (clone CY90; Serotec Ltd., Oxford, Oxfordshire, UK), epithelial membrane antigen (EMA) (clone E29; Dako UK Ltd.), CD31 (clone JC70A; Dako UK Ltd.), CD45 (clone 2B11/PD7/26; Dako UK Ltd.), alpha-smooth muscle actin (α-SMA) (clone 1A4; Dako UK Ltd.), and α1 integrin (clone SR84; BD Pharmingen, San Diego, CA, USA). Fluorescein isothiocyanate-conjugated rabbit anti-mouse F(ab^1^)_2 _was used as a secondary antibody, and staining was recorded on a Zeiss Axiovert 200 M confocal microscope using the LSM 510 Meta software (Carl Zeiss, Jena, Germany). Omission of the primary antibody was included as a negative control.

### Generation of fibroblast conditioned medium

Fibroblasts were plated into 25-cm^2 ^tissue culture flasks and grown to 60% confluency, the media removed, cells washed with Dulbecco's phosphate-buffered saline (dPBS), and 5 mL of serum-free DMEM was added. Fibroblasts then were cultured for a further 48 hours, and media were removed and centrifuged to remove cell debris and stored at -80°C until use. For the reproducibility assay, hfff_2 _cells were cultured in 75-cm^2 ^tissue culture flasks generating 15 mL of CM in order to allow assays to be performed in triplicate.

### Invasion assays

Two types of invasion assay were performed. In one, donor fibroblasts were incorporated into the lower well of the invasion assay (Fib co-culture assay), allowing crosstalk between the fibroblast and tumor cell populations. In the other, CM, generated from an equivalent number of fibroblasts, was placed in the lower well of the invasion assay. The invasion assays were carried out over the course of 48 hours as described previously [32]. Briefly, the lower surface of an 8-μm-pore polyethylene terephthalate track-etched membrane was coated with 10 μg/mL fibronectin (Sigma-Aldrich Company Ltd.) and the upper surface was coated with Englebreth-Holm-Swarm basement membrane (Matrigel; Becton, Dickinson and Company, Franklin Lakes, NJ, USA) at a final concentration of 5 μg per filter. The upper membrane was seeded with 5 × 10^4 ^MDA-MB-468 cells in serum-free DMEM, and either 1 × 10^5 ^fibroblasts or fibroblast CM (1 mL) was placed into the lower chamber. Invasion assays were set up in quadruplicate, cell counts were performed on hematoxylin and eosin-stained membranes, and an invasion index was calculated as a percentage of the number of cells on the lower membrane compared with the total number of cells (on the upper and lower membranes).

For invasion assays in which human recombinant MMP-3 was included (R&D Systems Europe Ltd, Abingdon, Oxfordshire), a range of MMP-3 concentrations (12.5 to 100 ng/mL) was added at the start of the assay. For invasion assays incorporating inhibitors, 25 μM MMP-3 inhibitor (Ac-RCGVPD-NH_2_; code number: 444218; Calbiochem, now part of EMD Biosciences, Inc., San Diego, CA, USA) or vehicle control was added at the beginning of the assay and again at 24 hours. The assays then were processed as described above. At the end of the invasion assay, medium from the four wells of the assay was harvested (referred to as end-of-assay CM), centrifuged to remove cell debris, and stored at -80°C until use.

### Analysis of MMP polymorphism status

Purification of genomic DNA from frozen fibroblast populations was carried out using the DNA Research Innovations Ltd Genomic DNA purification kit (DNA Research Innovations Ltd, Kent, UK) according to the manufacturer's instructions. Briefly, approximately 250,000 cells in 100 μL of dPBS was lysed in lysis buffer containing proteinase K for 5 minutes and DNA was captured using magnetic beads with a DNA-binding positive charge. DNA was eluted from the beads using a low-salt (10 mM Tris-HCl; pH 8.5) buffer. DNA concentration and purity were assessed by means of a spectrophotometer. Fibroblast populations from 19 normal donors and 13 patients with breast cancer were analyzed. As an internal control for the MMP polymorphism polymerase chain reaction (PCR), nine paired dermal samples also were included in the study.

PCR was performed using 100 ng of DNA in each reaction with the following primers: *MMP-1 *forward: TCG TGA GAA TGT CTT CCC ATT, *MMP-1 *reverse: TCT TGG ATT GAT TTG AGA TAA GTG AAA TC [32]; *MMP-3 *forward: GAT TAC AGA CAT GGG TCA CA, *MMP-3 *reverse: TTT CAA TCA GGA CAA GAC GAA GTT T [33]; and *MMP-9 *forward: GCC TGG CAC ATA GTA GGC CC, *MMP-9 *reverse: CTT CCT AGC CAG CCG GCA TC [34]. PCR products were digested at 37°C for 18 hours using 1 U per reaction *xml-1 *(*MMP-1 *and *MMP-3*) or *sphI *(*MMP-9*) (New England Biolabs, Inc., Ipswich, MA, USA), and digestion products were analyzed using agarose gel electrophoresis.

### Measurement of MMP release and activity

Substrate gel zymography was carried out as described previously [35]. The end-of-assay CM was concentrated using Centricon centrifugal filter devices (YM-10; Millipore Corporation, Billerica, MA, USA) and 50 μg of protein mixed with 10× non-reducing sample buffer (0.3 M Tris-HCL [pH 6.8], 12.5% glycerol, 1% SDS, and 1% bromophenol blue). Samples were run on a 12% polyacrylamide gel containing 0.01% SDS and a final concentration of 1 mg/mL gelatin. Recombinant pro-MMP-2 and pro-MMP-9 (Calbiochem) were included as standards on each gel. Electrophoresis was carried out for 3 hours at 120 V before renaturation of proteins by three 15-minute washes in 2.5% Triton X-100. Gels were incubated overnight at 37°C in developing buffer (0.5 M Tris, 2 M NaCl, 0.05 M CaCl_2_, and 0.2% Triton X-100) and stained with 0.5% Coomassie Blue. Gelatinolytic activity was visualized, as clear bands on the uniformly blue-stained background, and quantitated by densitometry. A sandwich enzyme immunoassay kit (R&D Systems Europe Ltd, Abingdon, Oxfordshire) was used to measure total MMP-3 present in end-of-assay CM and was carried out according to the manufacturer's instructions.

### RT-PCR analysis of MMP expression

To determine the cellular source of MMP, RNA was isolated from cell populations and reverse transcription (RT)-PCR was carried out for *MMP-1*, *MMP-3*, and the housekeeping gene *GAPDH*. Total RNA was extracted using a standard phenol/chloroform extraction procedure [36]. After quantitation, 1 μg of total RNA was taken from each sample and cDNA was generated using the Promega Reverse Transcription kit according to the manufacturer's instructions. PCR was carried out using megamix reaction buffer (Microzone Limited, Haywards Heath, West Sussex, UK), 1 μL of cDNA in each reaction, and 200 nM of primer. Primer sequences were as follows: *GAPDH *forward: AGA ACA TCA TCC CTG CCT CC, *GAPDH *reverse: GCC AAA TTC GTT GTC ATA CC; *MMP-1 *forward: CGA CTC TAG AAA CAC AAG AGC AAG A, *MMP-1 *reverse: AAG GTT AGC TTA CTG TCA CAC GCT T; and *MMP-3 *forward: TCT GAA AGT CTG GGA AGA GGT C, *MMP-3 *reverse: CAG TGT TGG CTG AGT GAA AGA G. PCR products were analyzed on a 2% agarose gel.

### Statistical analysis

Statistical analysis was performed using the SPSS 12.0 statistics package (SPSS Inc., Chicago, IL, USA). For comparison between normal breast and tumor donor samples, an independent samples test was carried out. Odds ratios (ORs) and 95% confidence intervals (CIs) were calculated as an index of the association of genotype and donor group. For comparison of within-donor variables, such as CM assay versus Fib assay and breast versus dermal IPC, a repeated-measures analysis-of-variance analysis was performed. All statistical tests were two-sided, and a *p *value of less than 0.05 was considered significant.

## Results

### Isolation and characterization of fibroblasts

Fibroblasts were isolated from 32 donors: 13 with invasive breast carcinoma and 19 with no breast pathology. In the tumor donor cases, the fibroblasts were isolated from within the carcinoma (that is, tumor-associated fibroblasts, or TAFs).

**Figure 1:**
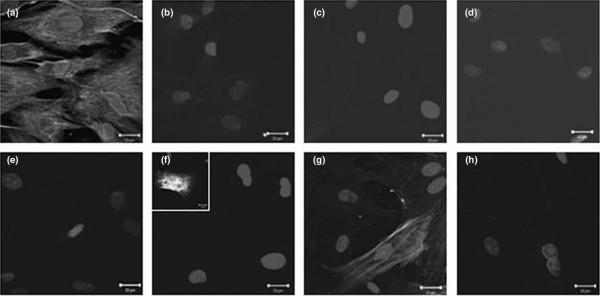
Characterization of isolated fibroblast populations. Isolated breast fibroblast populations were characterized by immunofluorescent staining. All cells exhibited staining for vimentin **(a) **but were uniformly negative for the luminal epithelial membrane antigen **(b)**, cytokeratin 18 **(c)**, the endothelial-associated CD31 **(d)**, and pericyte-related α_1_-integrin **(e)**. Approximately 1% of cells stained strongly for cytokeratin 14 **(f, inset)**, and approximately 35% of cells were positive for alpha-smooth muscle actin **(g)**. **(h) **Negative control.

The purity of the fibroblast populations was confirmed by immunostaining. The isolated cells (>95%) stained uniformly for vimentin and were negative for the luminal epithelial-associated EMA and CK18, the endothelial-associated CD31, and the pericyte and vascular smooth muscle-related α1-integrin (Figure 1a–e). The isolated cells also were negative for the leukocyte marker CD45 (not shown). The majority of the cells were negative for the myoepithelial-associated cytokeratin CK14. However, approximately 1% of cells exhibited strong staining for this cytokeratin (Figure 1f). A similar pattern of reactivity was seen in the hfff_2 _cell line, suggesting that this staining pattern does not represent contaminating myoepithelial cells. Cell populations also were stained for α-SMA (Figure 1g) and this showed that approximately 35% of cells were positive at passage 4 when they were used in experiments, with no differences in populations discerned between TAFs and normal breast fibroblasts (data not shown). Taken together, the results indicate that the isolated fibroblast populations were substantially pure (>95%) and not contaminated with epithelial, myoepithelial, endothelial, inflammatory, or pericyte cell populations.

### Tumor-fibroblast interactions determine invasion-promoting capacity

**Figure 2:**
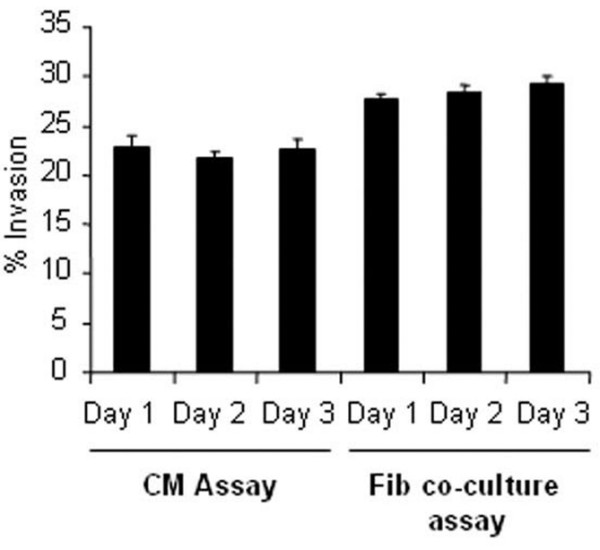
Reproducibility of invasion assays. Conditioned media (CM) and fibroblast co-culture invasion assays with the MDA MB 468 breast cancer cell line were carried out in triplicate on three separate days. A high level of reproducibility was obtained between experiments. The two different types of assay demonstrate significantly different levels of invasion, with higher tumor invasion in the presence of fibroblasts compared with CM alone (*p *= 0.001).

The reproducibility of the invasion assay was confirmed by using the hfff_2 _fibroblasts, or CM from these fibroblasts, with the MDA MB 468 breast cancer cell line in experiments conducted on three separate days. The results demonstrate a high level of reproducibility between assays with mean invasions of 22.2% (± 0.6%) in the CM assay and 28.4% (± 0.4%) in the fibroblast co-culture assay (Figure 2). The higher level of invasion generated in the co-culture assay, though modest in absolute terms, statistically was highly significant (*p *= 0.001) and suggests that the concomitant presence of fibroblasts is more stimulatory for invasive activity than conditioned medium alone.

**Figure 3:**
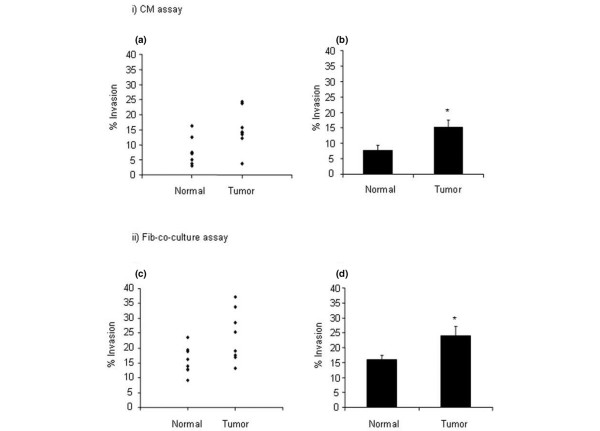
Relationship between fibroblast invasion-promoting capacity and donor type. Conditioned media (CM) (i) and fibroblast co-culture (ii) invasion assays were carried out comparing eight normal donors and eight tumor donors. Graphs **(a) **and **(c) **show scatterplots of individual donors, and **(b) **and **(d) **illustrate mean invasion generated by the two donor groups. In the CM assay, significantly higher levels of tumor invasion were generated by CM from tumor-derived fibroblasts compared with normal fibroblasts **(b) **(*p *= 0.009). Higher levels of invasion were generated in the fibroblast co-culture assays compared with the CM assay for both normal (*p *≤ 0.001) and tumor-derived (*p *= 0.02) fibroblasts **(b,d)**, and a significant difference between normal and tumor-derived fibroblast invasion-promoting capacity was demonstrated in the co-culture assay **(d) **(*p *= 0.039). * p =< 0.05.

Fibroblasts from normal breast were compared with fibroblasts from carcinomas in order to determine whether they had differing effects on stimulating tumor cell invasion. As shown in Figure 3, when the normal fibroblasts (*n *= 8) were used to create CM (as compared to the tumor fibroblasts; *n *= 8) and then assessed for stimulation of tumor cell invasion, there was variability between individual donors (Figure 3a). However, the mean percentage of tumor invasion was 7.7% (range 2.9% to 16.2%) after treatment with CM from normal fibroblasts versus 15.2% (range 3.8% to 24.3%) with CM from tumor fibroblasts (Figure 3b). These values were statistically different (*p *= 0.009) and indicate that CM from tumor-derived fibroblasts has a higher intrinsic IPC than CM from normal fibroblasts.

When fibroblasts were co-cultured with the tumor cells, considerable variability again was demonstrated between individual donors (Figure 3c). As in the reproducibility assays (Figure 2) for both normal fibroblasts and tumor-derived fibroblasts, higher levels of tumor invasion were generated in the presence of the fibroblast populations compared with CM (Figure 3b,d), with a mean invasion generated by normal fibroblasts of 15.8% (range 9.1% to 23.6%) compared with 7.7% (range 2.9% to 16.2%) with CM (*p *≤ 0.001) and with a mean invasion of tumor-derived fibroblasts of 24.0% (range 13.3% to 37.1%) compared with 15.2% (range 3.8% to 24.3%) with CM (*p *= 0.02). In both the CM assay and the co-culture assays, the presence of tumor fibroblasts generated significantly higher IPC than normal fibroblasts (*p *= 0.02) (Figure 3c,d). Since, physiologically, fibroblasts maintain interactions with the tumor cells, the fibroblast co-culture assays were used for further analysis.

### Tumor-derived fibroblast IPC, but not normal fibroblast IPC, relates to MMP polymorphism status

To assess the relationship between fibroblast IPC and MMP polymorphism status, donor DNA (*n *= 32) was analyzed for functional SNPs in *MMP-1*, *MMP-3*, and *MMP-9 *genes. The analysis was performed both on breast fibroblasts and on isolated skin fibroblasts in order to confirm genotype and exclude false calls resulting from possible loss of heterozygosity. In all cases, there was concordance between breast and skin DNA, and genotype was confirmed by sequencing (data not shown).

**Figure 4:**
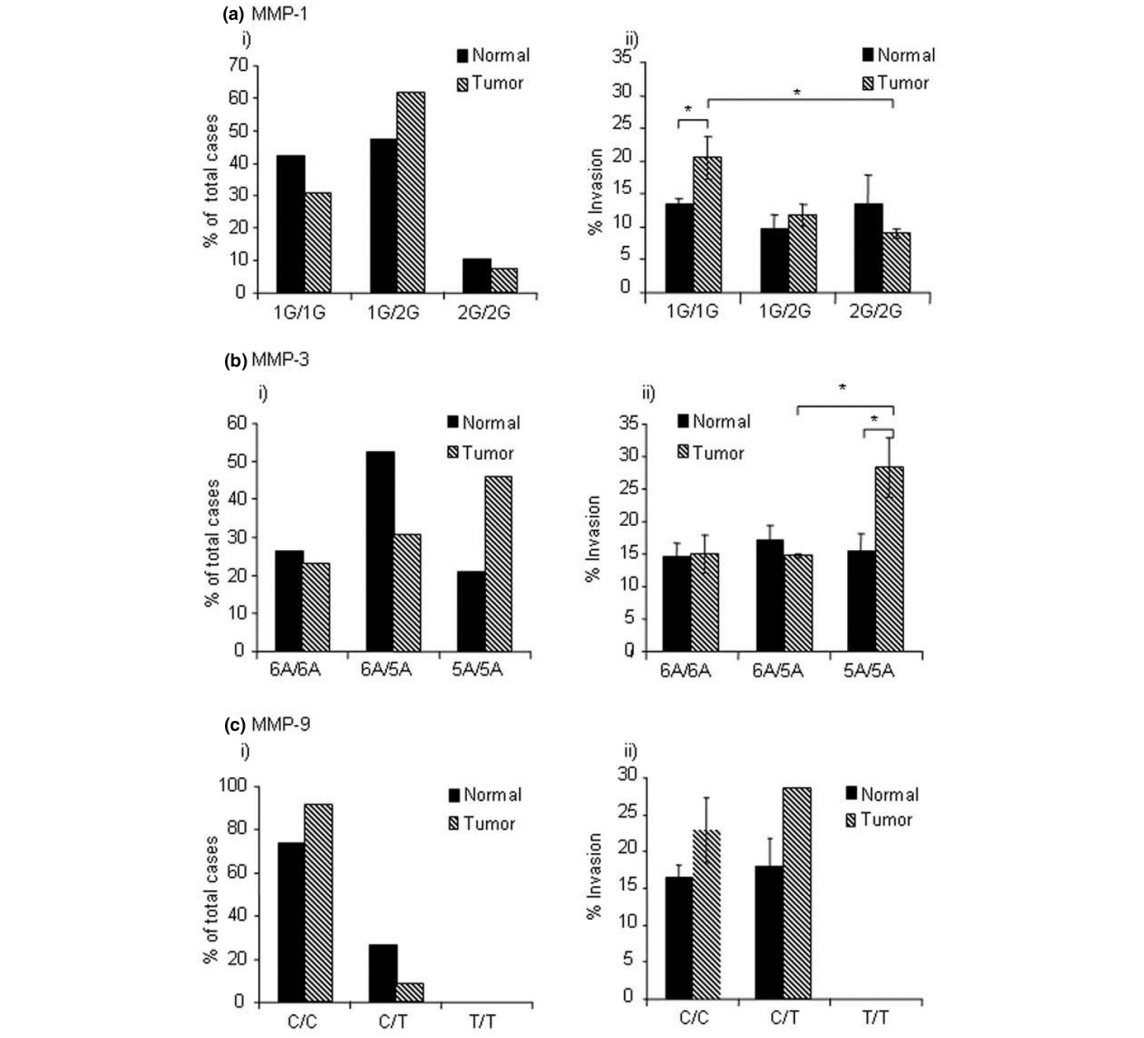
Relationship between fibroblast matrix metalloproteinase (MMP) polymorphism status and (i) donor type and (ii) invasion-promoting capacity. Donors (*n *= 19 normal, 13 tumor) were analyzed for MMP-1 **(a)**, MMP-3 **(b)**, and MMP-9 **(c) **polymorphism status. No significant relationship was identified between MMP genotype and donor type (i), although a trend toward increased frequency of the *MMP-3 *5A/5A genotype is observed in the breast cancer donor group **(a.i)**. When compared to fibroblast invasion-promoting capacity, higher tumor invasion was generated by donors with the *MMP-1 *1G/1G genotype compared with the 1G/2G and 2G/2G genotype **(a.ii) **(*p *= 0.08 and 0.032, respectively), and significantly higher invasion was promoted by tumor fibroblasts with the 1G/1G genotype compared with normal donors with the same genotype (*p *= 0.025). In addition, higher invasion was stimulated by fibroblasts with the *MMP-3 *5A/5A genotype compared with the 5A/6A or 6A/6A genotypes **(b.ii) **(*p *= 0.05 and 0.07, respectively), and significantly higher invasion was promoted by tumor donors with the 5A/5A genotype when compared with normal donors with the same genotype (*p *= 0.04). No relationship was identified between *MMP-9 *genotype and invasion-promoting capacity **(c.ii)**. * p =< 0.05

When genotype status was compared with donor type, no significant difference in SNP status for *MMP-1*, *MMP-3*, or *MMP-9 *was seen in tumor donors versus non-tumor donors (Figure 4), although there was a trend toward higher frequency of the *MMP-3 *5A/5A genotype amongst the breast cancer donor group, with the OR of relative risk associated with 5A/5A compared to the other two genotypes being 3.21 (95% CI 0.68 to 15.16; *p *= 0.14).

When MMP SNP status was compared to normal fibroblast IPC, no relationship was observed between genotype and IPC. However, in tumor-derived fibroblasts, there was an inverse relationship with *MMP-1 *genotype, with lower levels of invasion generated by donors with the high-expressing 2G/2G genotype compared with the 1G/2G or 1G/1G genotypes (*p *= 0.08 and 0.03, respectively) (Figure 4a.ii). Furthermore, tumor donors with the high-expressing *MMP-3 *5A/5A genotype exhibited higher IPC compared with 6A/5A and 6A/6A donor genotypes, (*p *= 0.05 and 0.07, respectively) (Figure 4b.ii), although no relationship was demonstrated between *MMP-9 *genotype and donor IPC (Figure 4c.ii). Significantly higher invasion was promoted by tumor fibroblasts with the *MMP-1 *1G/1G genotype compared with their normal counterparts (*p *= 0.025) (Figure 4a.ii) and, similarly, tumor fibroblasts with the *MMP-3 *5A/5A genotype demonstrated significantly higher IPC than normal fibroblasts with the same genotype (*p *= 0.04) (Figure 4b.ii). Donor numbers were too small to carry out formal linkage analysis. However, 6 of 10 donors with the *MMP-3 *5A/5A genotype also carried the *MMP-1 *1G/1G genotype.

### *MMP-3 *polymorphism status in relation to MMP expression and activation

**Figure 5:**
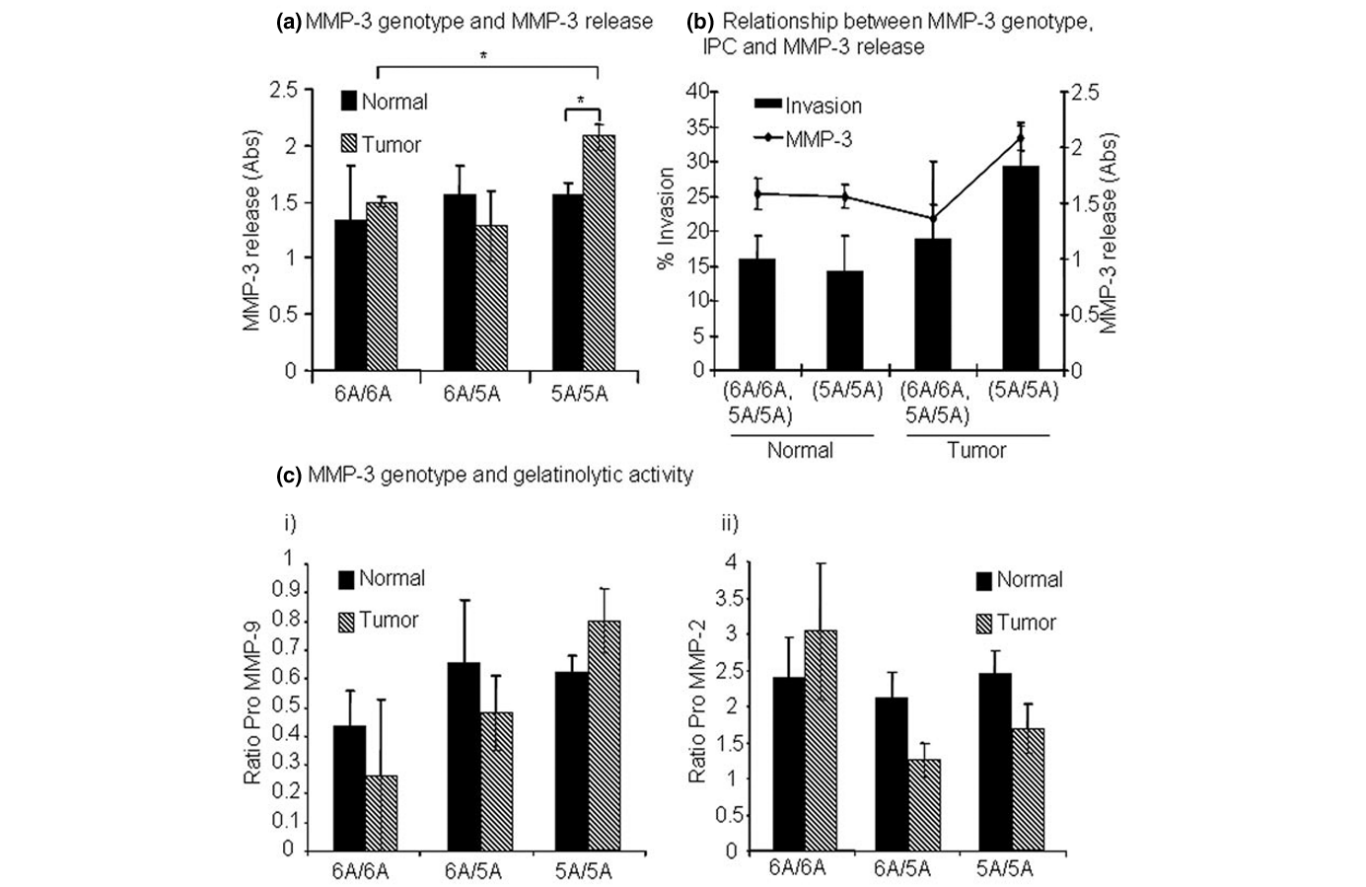
Relationship between matrix metalloproteinase-3 (MMP-3) polymorphism status and MMP release and activation. **(a) **Relationship between MMP-3 genotype and total MMP-3 release into end-of-assay conditioned media. The tumor fibroblast 5A/5A genotype is associated with higher levels of MMP-3 release compared with the tumor fibroblast 6A/5A genotype (*p *= 0.07) and the 6A/6A genotype (*p *= 0.009). Additionally, tumor fibroblasts with the 5A/5A genotype release significantly higher levels of MMP-3 than normal fibroblasts with this genotype. **(b) **Relationship between MMP-3 genotype, invasion-promoting capacity (IPC), and MMP-3 release. For tumor fibroblasts, there was a correlation between MMP-3 5A/5A genotype, a high IPC phenotype, and high MMP-3 release. **(c) **Relationship between MMP-3 genotype and (i) pro-MMP-2 and (ii) pro-MMP-9 levels measured by zymography. No significant association between genotype and gelatinolytic activity in normal or tumor fibroblasts was identified. Abs, antibodies. * p =< 0.05

The level of MMP-3 release in relation to SNP genotype was measured by enzyme-linked immunosorbent assay of end-of-assay CM. This indicated that tumor fibroblasts with the 5A/5A genotype secreted more MMP-3 than tumor fibroblasts with either the 6A/5A or 6A/6A genotypes (*p *= 0.07 and 0.009, respectively). In addition, tumor fibroblasts with the 5A/5A genotype were associated with significantly higher levels of MMP-3 release than normal fibroblasts with the 5A/5A genotype (*p *= 0.028) (Figure 5a) and this correlated also with a high IPC phenotype (Figure 5b).

Since MMP-3 can activate other MMPs involved in mediating tumor invasion, including MMP-9, the relationship between *MMP-3 *genotype, IPC, and gelatinase levels in end-of-assay CM was analyzed. No correlation was identified between MMP-2 or MMP-9 gelatinase levels and IPC or *MMP-3 *status (Figure 5c).

### Contribution of fibroblast-derived MMP-3 activity to tumor cell invasion

**Figure 6:**
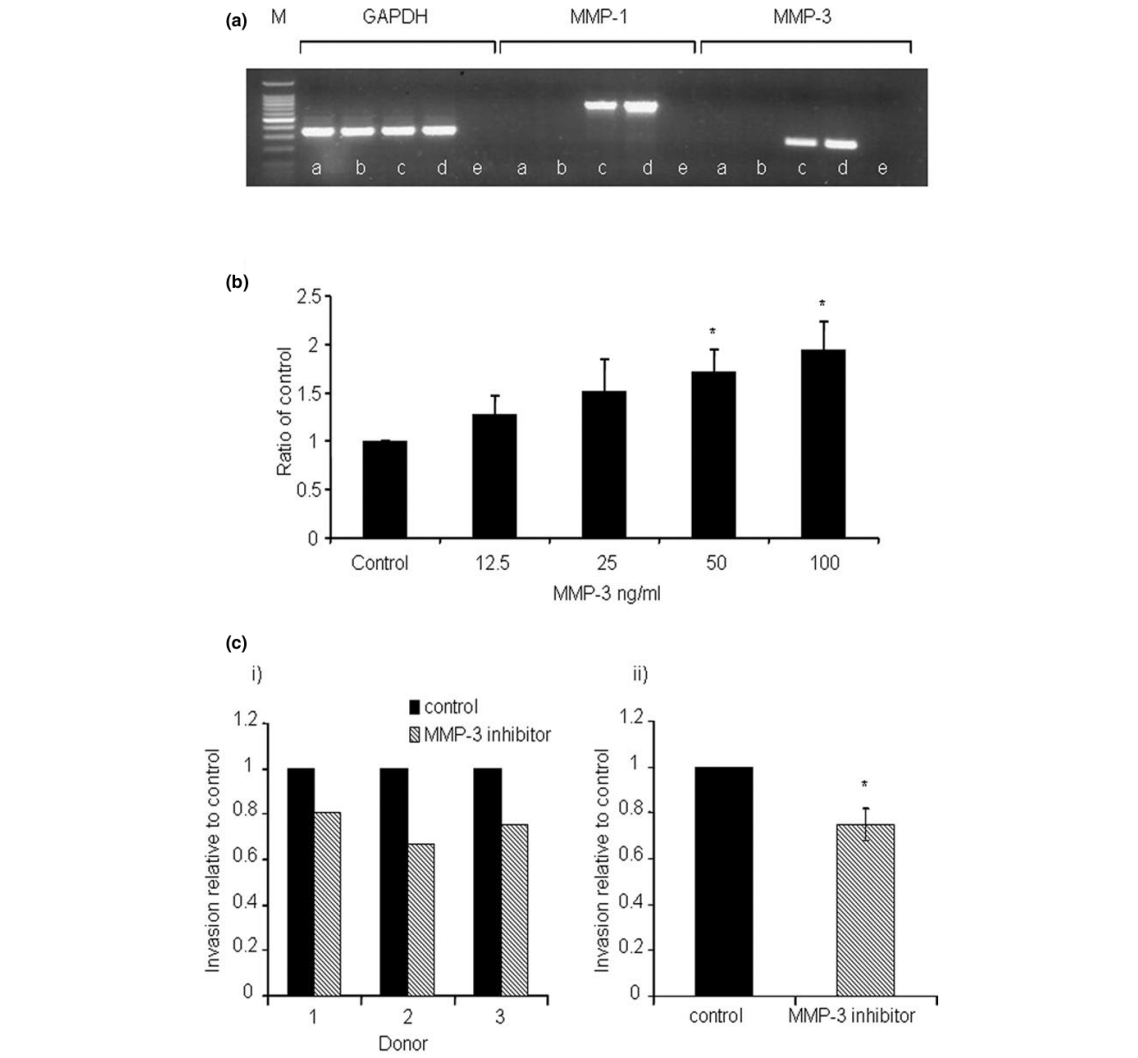
Contribution of fibroblast-derived matrix metalloproteinase-3 (MMP-3) activity to tumor cell invasion. **(a) **Reverse transcription-polymerase chain reaction for housekeeping gene *GAPDH*, MMP-1, and MMP-3 in tumor cell and fibroblast populations, indicating that MDA MB 468 tumor cells do not express MMP-1 or MMP-3, even following culture with fibroblast conditioned media (CM), whereas both primary fibroblasts and the hfff_2 _cell line are a source of these MMPs. (1) MDA MB 468; (2) MDA MB 468 + fibroblast CM; (3) hfff_2 _fibroblast cell line; (4) primary fibroblasts; (5) water blank. M, molecular weight markers. **(b) **Effect of human recombinant MMP-3 on invasion of MDA MB 468 cells. Increasing invasion is observed in the presence of increasing concentrations of MMP-3, with a doubling of invasion at 100 ng/mL MMP-3 compared with control (*p *= 0.014). **(c) **Effect of the MMP-3 inhibitor on fibroblast-stimulated tumor invasion: (i) shows tumor invasion generated by three separate donors, and (ii) shows mean invasion in the presence of MMP-3 inhibitor relative to control. There is a significant reduction in tumor cell invasion in the presence of MMP-3 inhibitor (*p *= 0.005). * p =< 0.05.

*In situ *hybridization studies suggest that the major source of MMPs in breast cancer is constituted by the stromal compartment [20,37]. To confirm that fibroblasts are the source of MMP in the current invasion assay systems, RT-PCR was carried out on the MDA MB 468 tumor cells cultured alone or following culture with fibroblast CM, and on primary breast fibroblasts and the hfff_2 _fibroblast cell line. This showed that the MDA MB 468 tumor cells used in the invasion assays in this study do not express *MMP-1 *or *MMP-3*, even following culture with fibroblast CM, whereas the fibroblast populations express both of these MMPs (Figure 6a).

### Contribution of MMP-3 activity to tumor cell invasion

To determine the contribution made by MMP-3 activity to tumor cell invasion, the invasion assays were carried out in the absence of MMP-3 (control) or the addition of a range of concentrations of human recombinant MMP-3. The concentrations used were determined by the levels of endogenous MMP-3 detected from the primary fibroblast cultures. The data demonstrated an incremental increase in tumor cell invasion with increasing concentration of MMP-3 (Figure 6b), with a doubling of invasion in the presence of 100 ng/mL MMP-3 compared with control (*p *= 0.014). To demonstrate the contribution of fibroblast-derived MMP-3, co-culture invasion assays were carried out in the presence of the specific MMP-3 inhibitor, Ac-RCGVPD-NH_2_. This revealed a consistent and highly significant reduction in tumor cell invasion in the presence of the MMP-3 inhibitor compared with vehicle-only control assays (*p *= 0.005) (Figure 6b), thus supporting a role for fibroblast-derived MMP-3 activity in promoting tumor cell invasion.

## Discussion

There is increasing recognition of the importance of the microenvironment in modulating tumor cell behavior [1,2,38], particularly of the role of fibroblasts in this process [5,9]. MMPs could play a multifaceted role in such a phenomenon, modifying the tumor microenvironment through direct breakdown of matrix proteins and basement membrane barriers, promotion of angiogenesis, and/or the release and activation of growth factors [18]. Both MMP-1 and MMP-*3 *have been shown to affect the motility and invasion of tumor cells directly [39,40]. Therefore, differences in the capacity to express MMPs may be critical in regulating tumor behavior. One of the mechanisms by which MMP expression could be modified is through the existence of functional SNPs capable of influencing gene promoter activity [23-26]. Several studies have examined the association between MMP SNP genotype and the prevalence of breast cancer [27-30], but directly relating such intrinsic characteristics to cellular behavior is difficult owing to the challenge of isolating primary cells for functional studies from a large enough group of donors. This is the first study to use purified cell populations from different donor groups in order to investigate the impact of MMP SNP genotype on tumor cell behavior. Since the major source of MMPs in breast cancer is the stromal fibroblast [20,22,37], we examined the relationship between MMP SNP genotype and the ability of donor fibroblasts to promote breast cancer cell invasion. The data show that, whereas fibroblasts from different donors vary in their capacity to promote tumor invasion, significantly higher levels of tumor invasion are generated by tumor fibroblasts compared with normal fibroblasts, results in keeping with previous studies [2,17,41]. This was seen both in CM and fibroblast co-culture assays, although the level of tumor invasion was higher in the presence of fibroblasts, indicating a role for tumor-fibroblast interactions in determining tumor behavior.

When fibroblast IPC was related to MMP SNP genotype, we identified a significant association between the high-expressing MMP-3 5A/5A genotype and high IPC of donor fibroblasts for the tumor-derived populations, though not in the normal fibroblast donors. Thus, whereas tumor-derived fibroblasts of MMP-3 5A/5A status exhibited significantly higher invasion than tumor fibroblasts of 5A/6A or 6A/6A status, the IPC of normal fibroblasts of MMP-3 5A/5A genotype did not differ from fibroblasts of 5A/6A or 6A/6A status. Furthermore, the *MMP-3 *5A/5A genotype and high IPC were reflected in higher levels of MMP-3 activity in tumor-derived fibroblasts, although the normal donor fibroblasts of *MMP-3 *5A/5A genotype did not exhibit enhanced MMP-3 activity. This suggests that tumor-derived fibroblasts have undergone additional changes that influence the phenotypic expression of the MMP SNP genotype and that the high IPC of tumor-derived fibroblasts is not simply a response to tumor-derived signals. It previously has been demonstrated that the tumor-promoting capacity of tumor fibroblasts is a stable feature of this population [2], and there are several reports of distinct genetic and epigenetic changes in TAFs [13-15]. It is plausible that epigenetic alterations, such as demethylation (which is common in tumors [42]), may unmask the high-expressing potential of the *MMP-3 *5A/5A genotype in tumor fibroblasts. Despite previous reports of the wide variation in expression detected in relation to SNPs [43,44], in our study the *MMP-3 *5A/5A genotype was reflected in significantly higher levels of MMP-3 release in patients with breast cancer compared with the 5A/6A genotype, with the difference not quite reaching significance for the 6A/6A genotype group.

In apparent contradiction to the predicted enhancing role of MMPs, there was an inverse relationship between the high-expressing *MMP-1 *2G/2G genotype and invasion. This possibly reflects the nature of the invasion barrier used in these assays; thus, we measured invasion through basement membrane, which would be degraded by MMP-3, rather than invasion through interstitial collagen, which would be more dependent on MMP-1 activity. Alternatively, the inverse relationship between IPC and the high-expressing *MMP-1 *2G/2G genotype may reflect linkage disequilibrium between the *MMP-1 *2G and *MMP-3 *6A alleles as has been reported in colorectal cancer [24]. The number of donors in this study, though large in comparison to other studies, was too small to perform linkage analysis, but the results do suggest a relationship between *MMP-1 *2G and *MMP-3 *6A, and formal analysis on a larger patient cohort currently is being undertaken.

Perhaps different MMPs are important at different stages of disease progression and the models used here are more representative of early-stage invasion. The importance of MMP-3 at this initial stage of invasion is reflected in animal models in which targeting overexpression of MMP-3 to normal mouse mammary glands has been shown to induce an altered stromal environment promoting the phenotypic conversion and malignant transformation of mammary epithelial cells [45,46]. Moreover, MMP-3, in addition to other MMPs, has been implicated in Wnt1-induced mouse mammary tumorigenesis [22]. Such results are consistent with the possibility that MMP-3 expression may be important in the initiation of breast cancer. The functional importance of MMP-3 activity was demonstrated by a significant reduction in tumor cell invasion in the presence of a selective MMP-3 inhibitor. Crucially, our results indicate a stronger relationship between fibroblast IPC and *MMP-3 *genotype than the fibroblast IPC and donor status since, in our system, tumor-derived fibroblasts of *MMP-3 *5A/6A or 6A/6A genotype did not differ in their IPC from normal fibroblasts. Thus, while tumor-derived fibroblasts are stimulatory of tumor invasion, whether or not they are of the *MMP-3 *5A/5A genotype is more important.

Evidence for a role for MMP-3 in breast cancer comes from tissue studies and animal models. *MMP-3 *expression is detected in the stroma around invasive breast tumors [20,22,47] and is an indicator of poor prognosis [21]. Interestingly, our study showed that fibroblasts derived from patients with breast cancer were more frequently of the *5A/5A *genotype compared with normal fibroblasts and, together, this supports a previous study suggesting an association between the *MMP-3 *5A SNP and breast cancer susceptibility [27], although two subsequent studies failed to find such an association [28,29]. All three studies, however, found an association with lymph node positivity in patients with breast cancer, which we interpret as being consistent with our findings of a direct involvement of MMP-3 in tumor invasion promotion.

As well as degrading a wide spectrum of matrix proteins, MMP-3 activates other MMPs, including MMP-9 [48]. This could further enhance tumor invasion. However, no clear or significant relationship between *MMP-3 *SNP status and MMP-3 release with MMP-9 gelatinolytic levels was identified in this study.

## Conclusion

This study has shown that tumor-fibroblast-derived MMP-3 release, associated with the *MMP-3 *5A/5A genotype, enhances tumor invasion, and it suggests that women with this genotype may suffer from enhanced tumor progression. This report emphasizes the importance of microenvironmental factors – and the intrinsic characteristics of the host microenvironment – in cancer evolution.

## Abbreviations

bp = base pairs; CI = confidence interval; CM = conditioned media; DMEM = Dulbecco's modified Eagle's medium; dPBS = Dulbecco's phosphate-buffered saline; EMA = epithelial membrane antigen; FBS = fetal bovine serum; IPC = invasion-promoting capacity; MMP = matrix metalloproteinase; OR = odds ratio; PCR = polymerase chain reaction; RT-PCR = reverse transcription-polymerase chain reaction; SMA = smooth muscle actin; SNP = single nucleotide polymorphism; TAF = tumor-associated fibroblast.

## Competing interests

The authors declare that they have no competing interests.

## Authors' contributions

DLH carried out experimental studies, helped design the study, interpret the results, and draft the manuscript. JLJ designed the study, interpreted the results, and drafted the manuscript. SH helped carry out experimental studies. JAS and RAW characterized samples and contributed to the writing of the manuscript. All authors read and approved the final manuscript.

## References

[B1] Howlett AR, Bissell MJ (1993). The influence of tissue microenvironment (stroma and extracellular matrix) on the development and function of mammary epithelium. Epithelial Cell Biol.

[B2] Orimo A, Gupta PB, Sgroi DC, Arenzana-Seisdedos F, Delaunay T, Naeem R, Carey VJ, Richardson AL, Weinburg RA (2005). Stromal fibroblasts present in invasive human breast carcinomas promote tumor growth and angiogenesis through elevated SDF-1/CXCL12 secretion. Cell.

[B3] Bissell MJ, Radisky D (2001). Putting tumors in context. Nat Rev Cancer.

[B4] Weaver VM, Petersen OW, Wang F, Larabell CA, Briand P, Damsky C, Bissell MJ (1997). Reversion of the malignant phenotype of human breast cells in 3 dimensional culture and in-vivo by integrin blocking antibodies. J Cell Biol.

[B5] Shekhar MP, Werdell J, Santner SJ, Pauley RJ, Taiy L (2001). Breast stroma plays a dominant regulatory role in breast epithelial growth and differentiation: implications for tumor development and progression. Cancer Res.

[B6] Kuperwasser C, Chavarria T, Wu M, Magrane G, Gray JW, Carey L, Richardson AL, Weinburg RA (2004). Reconstruction of functionally normal and malignant human breast tissues in mice. Proc Natl Acad Sci USA.

[B7] Bhowmick NA, Chytil A, Plieth D, Gorska AE, Dumont N, Shappell S, Washington MK, Neilson EG, Moses HL (2004). TGF-beta signalling in fibroblasts modulates the oncogenic potential of adjacent epithelia. Science.

[B8] Howe JR, Roth S, Ringold JC, Summers RW, Jarvinen HJ, Sistonen P, Tomlinson IP, Houlston RS, Bevan S, Mitros FA (1998). Mutations in the SMAD4/DPC4 gene in juvenile polyposis. Science.

[B9] Kalluri R, Zeisberg M (2006). Fibroblasts in cancer. Nat Rev Cancer.

[B10] Bhowmick NA, Neilson EG, Moses HL (2004). Stromal fibroblasts in cancer initiation and progression. Nature.

[B11] Allinen M, Beroukhim R, Cai L, Brennan C, Lahti-Domenici J, Huang H, Porter D, Hu M, Chin L, Richardson A (2004). Molecular characterisation of the Tumor microenvironment in breast cancer. Cancer Cell.

[B12] Lewis MP, Lygoe KA, Nystrom ML, Anderson WP, Speight PM, Marshall JF, Thomas GJ (2004). Tumour-derived TGF-beta 1 modulates myofibroblast differentiation and promotes HGF/SF-dependent invasion of squamous carcinoma cells. Br J Cancer.

[B13] Moinfar F, Man YG, Arnould L, Bratthauer GL, Ratschek M, Tavassoli FA (2000). Concurrent and independent genetic alterations in the stromal and epithelial cells of mammary carcinoma: implications for tumorigenesis. Cancer Res.

[B14] Fukino K, Shen L, Matsumoto S, Morrison CD, Mutter GL, Eng C (2004). Combined total genome loss of heterozygosity scan of breast cancer stroma and epithelium reveals multiplicity of stromal targets. Cancer Res.

[B15] Hu M, Yao J, Cai L, Bachman KE, van den Brule F, Velculescu V, Polyak K (2005). Distinct epigenetic changes in the stromal cells of breast cancers. Nat Genet.

[B16] Hanson JA, Gillespie JW, Grover A, Tangrea MA, Chuaqui RF, Emmert-Buck MR, Tangrea JA, Libutti SJ, Linehan WM, Woodson KG (2006). Gene promoter methylation in prostate tumor-associated stromal cells. J Natl Cancer Inst.

[B17] Schor SL, Schor AM (2001). Phenotypic and genetic alterations in mammary stroma: implications for tumor progression. Breast Cancer Res.

[B18] Hannan MA, Siddiqui Y, Rostom A, Al-Ahdal MN, Chaudhary MA, Kunhi M (2001). Evidence of DNA repair/processing defects in cultured skin fibroblasts from breast cancer patients. Cancer Res.

[B19] Egeblad M, Werb Z (2002). New functions for the matrix metalloproteinases in cancer progression. Nat Rev Cancer.

[B20] Heppner KJ, Matrisian LM, Jensen RA, Rodgers WH (1996). Expression of most matrix metalloproteinase family members in breast cancer represent a tumor-induced host response. Am J Pathol.

[B21] Duffy MJ, Maguire TM, Hill A, McDermott E, O'Higgins N (2000). Metalloproteinases: role in breast carcinogenesis, invasion and metastasis. Breast Cancer Res.

[B22] Blavier L, Lazaryev A, Dorey F, Shackleford GM, DeClerck YA (2006). Matrix metalloproteinases play an active role in wnt1-induced mammary tumorigenesis. Cancer Res.

[B23] Ye S, Eriksson P, Hamsten A, Kurkinen M, Humphries SE, Henney AM (1996). Progression of coronary atherosclerosis is associated with a common genetic variant of the human stromelysin-1 promoter which results in reduced gene expression. J Biol Chem.

[B24] Hinoda Y, Okayama N, Takano N, Fujimura K, Suehiro Y, Hamanaka Y, Hazama S, Kitamura Y, Oka M (2002). Association of functional polymorphisms of matrix metalloproteinases (MMP)-1 and MMP-3 genes with colorectal cancer. Int J Cancer.

[B25] Rutter JL, Mitchell TI, Buttice G, Meyers J, Gusella JF, Ozelius LJ, Brinckerhoff CE (1998). A single nucleotide polymorphism in the matrix metalloproteinase promoter creates an Ets binding site and augments transcription. Cancer Res.

[B26] Zhang B, Ye S, Herrmann SM, Eriksson P, de Maat M, Evans A, Arveiler D, Luc G, Cambien F, Hamsten A (1999). Functional polymorphism in the regulatory region of gelatinase B gene in relation to severity of coronary atherosclerosis. Circulation.

[B27] Ghilardi G, Biondi ML, Caputo M, Leviti S, DeMonti M, Guagnellini E, Scorza R (2002). A single nucleotide in the matrix metalloproteinase 3 promoter enhances breast cancer susceptibility. Clin Cancer Res.

[B28] Biondi ML, Turri O, Leviti S, Seminati M, Cecchini F, Bernini M, Ghilardi G, Guagnellini E (2000). MMP-1 and MMP-3 polymorphisms in promoter regions and cancer. Clin Chem.

[B29] Krippl P, Langsenlehner U, Renner W, Yazdani-Biuki B, Koppel H, Leithner A, Wascher TC, Paulweber B, Samonigg H (2004). The 5A/6A polymorphism of the matrix metalloproteinase 3 gene promoter and breast cancer. Clin Cancer Res.

[B30] Przybylowska K, Kluczna A, Zadrozny M, Krawczyk T, Kulig A, Rykala J, Kolacinska A, Morawiec Z, Drzewoski J, Blasiak J (2006). Polymorphisms in the promoter region of the matrix metalloproteinase genes MMP-1 and MMP-9 in breast cancer. Breast Cancer Res Treat.

[B31] Sternlicht MD, Werb Z (2001). How matrix metalloproteinases regulate cell behaviour. Annu Rev Cell Dev Biol.

[B32] Jones JL, Shaw JA, Pringle JH, Walker RA (2003). Primary breast myoepithelial cells exert an invasion-suppressor effect on breast cancer cells via paracrine down-regulation of MMP expression in fibroblasts and tumor cells. J Path.

[B33] Dunleavey L, Beyzade S, Ye S (2000). Rapid genotype analysis of the matrix metalloproteinase 1 gene 1G/2G polymorphism that is associated with risk of cancer. Matrix Biology.

[B34] Dunleavey L, Beyzade S, Ye S (2000). Rapid genotype analysis of the matrix metalloproteinase 3 gene 5A/6A polymorphism. Atherosclerosis.

[B35] Crowther M, Goodall S, Jones JL, Bell PR, Thompson MM (2000). Localisation of matrix metalloproteinase 2 within the aneurysmal and normal aortic wall. Br J Surg.

[B36] Chomczynski P, Sacchi N (1987). Single-step method of RNA isolation by acid guanidinium thiocyanate-phenol-chloroform extraction. Anal Biochem.

[B37] Poulsom R, Hanby AM, Pignatelli M, Jeffrey RE, Longcroft JM, Rogers L, Stamp GW (1993). Expression of gelatinase A and TIMP-2 mRNAs in desmoplastic fibroblasts in both mammary carcinomas and basal cell carcinomas of the skin. J Clin Path.

[B38] Roskelley CD, Bissell MJ (2002). The dominance of the microenvironment in breast and ovarian cancer. Semin Cancer Biol.

[B39] Boire A, Covic L, Agarwal A, Jacques S, Sherifi S, Kuliopulos A (2005). PAR1 is a matrix metalloproteinase-1 receptor that promotes invasion and tumorigenesis of breast cancer cells. Cell.

[B40] Lochter A, Galosy S, Muschler J, Freedman N, Werb Z, Bissell MJ (1997). Matrix metalloproteinase stromelysin-1 triggers a cascade of molecular alterations that leads to stable epithelial-to-mesenchymal conversion and a premalignant phenotype in mammary epithelial cells. J Cell Biol.

[B41] Lebret SC, Newgreen DF, Thompson EW, Ackland ML (2007). Induction of epithelial to mesenchymal transition in PMC42-LA human breast carcinoma cells by carcinoma-associated fibroblast secreted factors. Breast Can Res.

[B42] Szyf M, Pakneshan P, Rabbani SA (2004). DNA methylation and breast cancer. Biochem Pharmacol.

[B43] Yan H, Yuan W, Velculescu VE, Vogelstein B, Kinzler KW (2002). Allelic variation in human gene expression. Science.

[B44] Wyatt CA, Coon CI, Gibson JJ, Brinckerhoff CE (2002). Potential for the 2G single nucleotide polymorphism in the promoter of matrix metalloproteinase to enhance gene expression in normal stromal cells. Cancer Res.

[B45] Sternlicht MD, Lochter A, Sympson CJ, Huey B, Rougier J-P, Gray JW, Pinkel D, Bissell MJ, Werb Z (1999). The stromal proteinase MMP3/stromelysin-1 promotes mammary carcinogenesis. Cell.

[B46] Sternlicht MD, Bissell MJ, Werb Z (2000). The matrix metalloproteinase stromelysin-1 acts as a natural tumor promoter. Oncogene.

[B47] Vizoso FJ, Gonzalez LO, Corte MD, Rodriguez JC, Vazquez J, Lamelas ML, Junquera S, Merino AM, Garcia-Muniz JL (2007). Study of matrix metalloproteinases and their inhibitors in breast cancer. Br J Cancer.

[B48] Ramos-DeSimone N, Hahn-Dantona E, Sipley J, Nagase H, French DL, Quigley JP (1999). Activation of matrix metalloproteinase 9 (MMP-9) via a converging plasmin/stromelysin-1 cascade enhances tumor cell invasion. J Biol Chem.

